# Genes Required for the Fitness of Salmonella enterica Serovar Typhimurium during Infection of Immunodeficient *gp91*^−/−^ *phox* Mice

**DOI:** 10.1128/IAI.01423-15

**Published:** 2016-03-24

**Authors:** Andrew J. Grant, Olusegun Oshota, Roy R. Chaudhuri, Matthew Mayho, Sarah E. Peters, Simon Clare, Duncan J. Maskell, Pietro Mastroeni

**Affiliations:** a Department of Veterinary Medicine, University of Cambridge, Cambridge, United Kingdom; b Wellcome Trust Sanger Institute, Wellcome Trust Genome Campus, Hinxton, Cambridge, United Kingdom; University of California, Davis

## Abstract

Salmonella enterica causes systemic diseases (typhoid and paratyphoid fever), nontyphoidal septicemia (NTS), and gastroenteritis in humans and other animals worldwide. An important but underrecognized emerging infectious disease problem in sub-Saharan Africa is NTS in children and immunocompromised adults. A current goal is to identify Salmonella mutants that are not pathogenic in the absence of key components of the immune system such as might be found in immunocompromised hosts. Such attenuated strains have the potential to be used as live vaccines. We have used transposon-directed insertion site sequencing (TraDIS) to screen mutants of Salmonella enterica serovar Typhimurium for their ability to infect and grow in the tissues of wild-type and immunodeficient mice. This was to identify bacterial genes that might be deleted for the development of live attenuated vaccines that would be safer to use in situations and/or geographical areas where immunodeficiencies are prevalent. The relative fitness of each of 9,356 transposon mutants, representing mutations in 3,139 different genes, was determined in *gp91*^−/−^ *phox* mice. Mutations in certain genes led to reduced fitness in both wild-type and mutant mice. To validate these results, these genes were mutated by allelic replacement, and resultant mutants were retested for fitness in the mice. A defined deletion mutant of *cysE* was attenuated in C57BL/6 wild-type mice and immunodeficient *gp91*^−/−^ *phox* mice and was effective as a live vaccine in wild-type mice.

## INTRODUCTION

*S*almonella enterica causes systemic diseases (typhoid and paratyphoid fever), nontyphoidal septicemia, and gastroenteritis in humans and other animals worldwide. Salmonella enterica serovar Typhi and S. enterica serovar Paratyphi are restricted to humans and cause typhoid and paratyphoid fever, respectively. These are important febrile illnesses often seen in crowded and impoverished populations with inadequate sanitation that are exposed to unsafe water and food and in travelers visiting countries where these diseases are endemic (1). In 2010, typhoid and paratyphoid fevers together were estimated to account for 190,200 deaths (2). Mortality rates in untreated typhoid fever can be 10 to 15%. In some cases patients recover but remain carriers of the bacteria for many years. Invasive nontyphoidal salmonellae (iNTS) are estimated to cause over 2.1 million illnesses and 416,000 deaths in Africa annually despite antimicrobial treatment. Vaccine development for iNTS disease is a high priority (3). iNTS disease is linked with conditions which impair the immune system, such as a lack of antibodies in young children and multiple comorbidities, such as advanced HIV infection, malaria, hemolysis, sickle cell disease (SCD), malnutrition, and cytokine pathway deficiencies (4, 5).

Current measures for controlling Salmonella infections are far from ideal. The emergence of new multidrug-resistant strains has reduced the usefulness of most antimicrobials (6). Prevention by implementation of hygiene measures alone is proving insufficient. Currently licensed vaccines are far from optimal, and no effective vaccine or delivery system effective against NTS is available.

Live attenuated S. enterica vaccines are more effective in many different infection systems than nonliving ones due to their ability to generate protective T-helper 1 type immunity in addition to antibody responses (7). New live attenuated vaccines against Salmonella currently are being developed and tested (8). However, concerns remain about the use of live vaccines in general when these are targeted to areas where the diseases are endemic with a high incidence of conditions that impair the immune system, and there is reluctance to use live vaccines in animals bred for food production due to fears of side effects subsequent to the unlikely, but possible, spread to humans. Reversion to virulence would pose a serious threat to individuals with an impaired immune system who may inadvertently receive the live vaccine. Immunosuppression can be transient, latent, or undiagnosed, possibilities that are likely to be more frequent in children or in adults from countries where sophisticated diagnostic facilities are not always available.

We and others have found that some live attenuated Salmonella vaccines, such as aromatic-dependent (*aro*) mutants, *htrA* mutants, and *aroA htrA* double mutants, can cause lethal infections in immunodeficient mice such as those lacking in the production of reactive oxygen species (ROS), interleukin-12 (IL-12), or gamma interferon (IFN-γ) and in mice lacking T cells (9, 10). Therefore, bacterial mutants of these genes could be dangerous if used in patients with similar immune defects. We also have shown that other vaccines, specifically Salmonella pathogenicity island-2 (SPI-2) mutants of S. enterica, similar to the ones currently being tested in humans, regain virulence in ROS-deficient animals (11) and could prove dangerous in individuals with reduced efficiency of the oxygen-dependent antimicrobial mechanisms of phagocytes (e.g., chronic granulomatous disease [CGD] or malaria patients) (12, 13). These immunodeficiencies may very well be latent in the individual at the time of vaccination.

A current goal is to identify mutations that would allow the generation of live attenuated salmonellae that would not significantly regain virulence in the absence of key components of the immune system. The feasibility of this approach is supported by the evidence that some Salmonella mutants can retain this attenuation in situations of serious immunodeficiency in animal models of systemic infection. For example, SPI-2 mutants are attenuated in gene-targeted mice lacking IFN-γ, despite these strains being virulent in ROS-deficient animals (14).

Recent advances in high-throughput techniques to assess simultaneously the genotypes and relative fitness of individuals in complex pools of bacterial transposon (Tn) mutants allow us to utilize a genome-wide screen for genes necessary for bacterial fitness in the face of particular immunological challenges. Transposon-directed insertion site sequencing (TraDIS) is one such technique. TraDIS exploits very-high-throughput Illumina sequencing to obtain sequence reads directly from the region of DNA flanking each Tn insert in pools of mutants (15). This allows the location of the transposons to be precisely determined in a massively parallel manner. The number of reads obtained for each Tn allows the relative abundance of each mutant to be assessed, and a quantitative measure of fitness can be obtained by comparing data obtained before and after a selection step.

In the current study, we have used a stringent mouse model of immunodeficiency to perform a genome-wide screen with the potential to identify genes that can disable reversion to virulence in immunodeficient hosts and that therefore could be included in new generations of live vaccines against invasive salmonelloses.

## MATERIALS AND METHODS

### Bacterial strains, media, and growth conditions.

All wild-type (WT) strains, defined mutants, and plasmids are summarized in Table 1. The preparation of electrocompetent Escherichia coli and *S*. Typhimurium cells and transformations was performed as previously described (21). Bacteria were grown on Luria-Bertani (LB) medium. Media were supplemented with the appropriate antibiotic for selection (ampicillin, 100 μg/ml; kanamycin, 50 μg/ml; chloramphenicol, 10 μg/ml; tetracycline, 12.5 μg/ml).

**TABLE 1:** Bacterial strains and plasmids used in this study

Strain or plasmid	Relevant genotype or description^*a*^	Source and/or reference
*S*. Typhimurium		
SL1344	Virulent in mice with an i.v. LD_50_ of <20 CFU for innately susceptible mice	16
SL1344 *Mu* Tn library	Random Tn mutants; Kn^r^	17
SL1344 Tn*5* Tn library	Random Tn mutants; Kn^r^	17
SL1344 *aroC*	Δ*aroC*, Cm^r^	This study
SL1344 *aroC aroD htrA*	Δ*aroC*, Cm^r^; Δ*aroD*, Kn^r^; Δ*htrA*, Tc^r^	This study
SL1344 *aroC ssaV*	Δ*aroC*, Cm^r^; Δ*ssaV*, Kn^r^	This study
SL1344 *cydC*	Δ*cydC*, Cm^r^	This study
SL1344 *cydD*	Δ*cydD*, Cm^r^	This study
SL1344 *cydC cydD*	Δ*cydC* Δ*cydD*, Cm^r^	This study
SL1344 *cysE*	Δ*cysE*, Cm^r^	This study
SL1344 *dksA*	Δ*dksA*, Cm^r^	This study
SL1344 *ftsK*	Δ*ftsK*, Cm^r^	This study
SL1344 *miaA*	Δ*miaA*, Cm^r^	This study
SL1344 *nuoK*	Δ*nuoK*, Cm^r^	This study
SL1344 *nuo* operon	Δ*nuo* operon, Cm^r^	This study
SL1344 *ptsI*	Δ*ptsI*, Cm^r^	This study
SL1344 *recD*	Δ*recD*, Cm^r^	This study
SL1344 *secG*	Δ*secG*, Cm^r^	This study
SL1344 *seqA*	Δ*seqA*, Cm^r^	This study
SL1344 *sucA*	Δ*sucA*, Cmr	This study
SL1344 *suc* operon	Δ*suc* operon, Cm^r^	This study
SL1334 *thdF*	Δ*thdF*, Cm^r^	This study
SL1344 *tol* operon	Δ*tol* operon, Cm^r^	This study
SL1344 *tol pal* operon	Δ*tol pal* operon, Cm^r^	This study
SL1344 *yqiC*	Δ*yqiC*, Cm^r^	This study
*E. coli*		
DH5α	Subcloning efficiency DH5α competent cells; F^−^ Φ80*lacZ*ΔM15 Δ(*lacZYA-argF*) *U169 recA1 endA1 hsdR17*(r_K_^−^ m_K_^+^) *phoA supE44 thi-1 gyrA96 relA1* λ^−^	Thermo Fisher Scientific
Plasmids		
pACYC177	Ap^r^, Kn^r^	New England Biolabs, 18
pACYC184	Cm^r^, Tc^r^	New England Biolabs, 18
pBR322	Ap^r^, Tc^r^	New England Biolabs, 19
pBADλRED	Ap^r^	20

a LD_50_, 50% lethal infectious dose.

### Recombinant DNA techniques.

Standard methods were used for molecular cloning (22). Chromosomal and plasmid DNA purification and routine DNA modifications, including restriction endonuclease digestion of DNA, modifications of DNA, and ligations, were carried out using commercial kits and supplies according to the manufacturers' instructions (Qiagen, Thermo Fisher Scientific, and New England BioLabs). DNA concentration and purity were measured using a NanoDrop ND-1000 spectrophotometer. PCR primers (see Table S1 in the supplemental material) were designed using Primer3 (http://frodo.wi.mit.edu/) and purchased from Sigma (Sigma-Genosys, United Kingdom). PCRs were performed in 25-μl reaction volumes in 0.2 ml Eppendorf tubes in a PerkinElmer Gene Amp 2400 thermal cycler. Reaction mixtures contained 200 μM deoxynucleoside triphosphates (dNTPs), 2 mM Mg^2+^, 0.01 volume of Proof Start DNA polymerase (2.5 U μl^−1^; Qiagen), 0.1 volume of polymerase buffer (10×), 1 μM forward and reverse primers, and template DNA (∼50 ng plasmid DNA or ∼100 ng chromosomal DNA). Thermal cycler conditions were 94°C for 10 min and then 35 cycles of 94°C for 1 min, 55°C for 1 min, and 72°C for 1 min, followed by a final extension at 72°C for 10 min.

### Generation of defined mutants of *S*. Typhimurium.

Mutants were generated using a modification of the ET cloning procedure (23, 24) as previously described (20). PCR was used to amplify the chloramphenicol resistance cassette from pACYC184 (18), the kanamycin resistance cassette from pACYC177 (18), or the tetracycline resistance cassette from pBR322 (19) with 5′ and 3′ 60-bp homology arms complementary to the flanking regions of the gene to be deleted. Approximately 1 μg of linear PCR product was used for integration into the chromosome using a modification of the Lambda Red method (25), as previously detailed (26). Transformants were selected by plating onto medium containing chloramphenicol, kanamycin, or tetracycline as appropriate. Screening for the loss of the pBADλred helper plasmid was as previously described (27), using MAST ID Intralactam circles (MAST Diagnostics, Bootle Merseyside, United Kingdom) to screen for the absence of beta-lactamase in bacterial colonies. (Further details of the construction of mutants are provided in the supplemental material).

### Mouse infections.

All animals were handled in strict accordance with good animal practice as defined by the relevant international (Directive of the European Parliament and of the Council on the Protection of Animals Used for Scientific Purposes, Brussels 543/5) and local (Department of Veterinary Medicine, University of Cambridge) animal welfare guidelines. All animal work was approved by the ethical review committee of the University of Cambridge and was licensed by the United Kingdom Government Home Office under the Animals (Scientific Procedures) Act 1986. Sex- and aged-matched 9- to 12-week-old C57BL/6 wild-type mice (Harlan Olac Ltd.) and *gp91*^−/−^ *phox* mice (bred at the Wellcome Trust Sanger Institute) were infected by intravenous (i.v.) injection of bacterial suspensions in a volume of 0.2 ml. Statically grown cultures of wild-type *S*. Typhimurium SL1344 and defined mutants were grown overnight at 37°C from single colonies in 10 ml LB broth and then diluted in phosphate-buffered saline (PBS) to the appropriate concentration for inoculation. Bacterial cultures for the pools of Tn*5* and *Mu* Tn mutants (TraDIS pools) were grown as described previously (14). Inocula were enumerated by plating dilutions onto LB agar plates. Mice were killed by cervical dislocation, and the livers and spleens were aseptically removed and homogenized in sterile water using a Colworth Stomacher 80. The resulting homogenate was diluted in a 10-fold series in PBS, and LB agar pour plates were used to enumerate viable bacteria.

### TraDIS analysis of *S*. Typhimurium mutant pools.

The Illumina sequence reads from the input (bacteria inoculated into mice) and output (bacteria recovered from infected livers and spleens) pools (ENA no. ERA201074) were generated using single-end short-read sequencing with Tn-specific primers and by massively parallel sequencing of Tn-flanking regions in the input and output mutant pools. Genomic DNA was prepared and 2 μg fragmented by Covaris to an average size of ∼300 bp. Following end repair and A tailing, a modified Illumina adapter, synthesized and annealed by Integrated DNA Technologies (IDT) using oligonucleotides SplA5_top and SplA5_bottom (see Table S1 in the supplemental material), was ligated to the fragments for 40 min at 20°C. Ligated fragments were cleaned using Ampure XP beads (Beckman Coulter) with a bead-to-sample ratio of 0.8 to 1. PCR enrichment of fragments containing the Tn was performed using a primer homologous to each end of the Tn (Mu_AG_5′PCR, Mu_AG_3′PCR, Tn5_AG_5′PCR, or Tn5_AG_3′PCR; see Table S1) in conjunction with an adapter-specific primer containing an index tag (SplAP5.x; see Table S1). PCR products were cleaned using Ampure XP beads (Beckman Coulter), quantified by quantitative PCR (qPCR), and then pooled. The resultant products were sequenced on a HiSeq2500 (Illumina) using a specially modified recipe to overcome difficulties generated by the monotemplate Tn sequence. Briefly, a Tn-specific sequencing primer (Mu_AG_5′seq, Mu_AG_3′seq, Tn5_AG_5′seq, or Tn5_AG_3′seq; see Table S1) anneals to the Tn 10 bases away from the genomic DNA junction. Sequencing takes place with no imaging for the first 10 cycles followed by imaging for the next 40 cycles. This gives a 40-bp genomic DNA read. The template is denatured, the same sequencing primer is reannealed, and 10 cycles of sequencing takes place to give a 10-bp Tn read.

### Sequence analysis.

Illumina reads generated from the genomic DNA of the input and output pools containing Tn*5* and *Mu* Tn insertions were stripped of the 5′ Tn tags using Cutadapt version 1.8.1 (28), and the remainder of each sequence read was mapped to the *S*. Typhimurium SL1344 chromosome and plasmid sequences (GenBank accession numbers FQ312003, HE654724, HE654725, and HE654726) using BWA mem version 0.7.12-r1039 (29). The raw input and output read counts were loaded into R, version 3.0.2. A step was performed to eliminate nonspecific background reads by filtering out the insertions with a total read count across all samples of <100 reads. DESeq2 version 1.4.5 (30) was used to compare the reads mapped to specific insertion positions in the input pools and the corresponding insertion positions in the output pools. Following normalization to account for differential sequence coverage between samples, dispersions in the replicate samples were estimated, and a negative binomial distribution model implemented in DESeq2 was used to test the hypothesis that fitness score was equal to zero under the assumption that a particular mutant was present at equivalent levels of sequence coverage in the input and output pools. We obtained *P* values for all mutants, and the *P* values then were adjusted for multiple testing using the Benjamini and Hochberg false discovery rate (31).

A fitness score for each Tn mutant was calculated as a log_2_ transformation of the ratio of normalized output-input read counts. Hence, the calculated fitness scores (log_2_-fold change) represent the difference in abundance of each mutant in the output pool relative to the input. We defined significantly attenuated mutants as those with a negative log_2_-fold change and an adjusted *P* value (Benjamini-Hochberg) of ≤0.05.

For comparative purposes, the sequence data from our previous study investigating the survival of the same Tn mutants in BALB/c mice (32) were analyzed using the same methods as those described above, and the corresponding mutants in the two data sets (i.e., those with reads mapping to the same genomic locus in the same orientation) were identified.

## RESULTS AND DISCUSSION

### *S*. Typhimurium TraDIS infections in C57BL/6 wild-type and *gp91*^−/−^ *phox* mice.

Gp91 is one of the subunits of the NADPH oxidase essential for ROS production by phagocytes. ROS deficiency becomes apparent from the very early stages of an S. enterica infection, leading to a reduction in initial killing of the bacteria and accelerated growth in the first few days of infection. Therefore, a short course of infection (48 h) was required to investigate the behavior of *S*. Typhimurium mutants in the *gp91*^−/−^ *phox* mice.

We screened 21 pools of 480 Tn mutants of *S*. Typhimurium in each pool, covering ∼10,000 Tn*5* and *Mu* Tn mutants, at a dose of ∼5 × 10^4^ log_10_ CFU (the mean input dose in each of the 21 pools was 4.65 ± 0.04 log_10_ CFU; errors are given as standard deviations) in duplicate 8-week-old C57BL/6 wild-type (WT) or *gp91*^−/−^ *phox* mice. The dose and pool size were dictated by the need to allow the progression of the infection until the 48-h time point without it reaching lethal bacterial numbers. We administered pools of *S*. Typhimurium Tn mutants via the parenteral i.v. route in order to relate our outputs to those of genes and mechanisms of immunity involved in the control of the systemic phase of an invasive infection. The control of growth at this systemic level (i.e., restraint of bacterial growth and dissemination) is an absolute requirement for survival in S. enterica typhoidal and septicemic infections in several target hosts (32).

Mice were killed at 48 h postinfection (p.i.) when the mean bacterial load per liver was, on average, 2.90 log_10_ CFU greater in the *gp91*^−/−^ *phox* mice than in C57BL/6 WT mice (*gp91*^−/−^ *phox* mice, 8.51 ± 0.33 log_10_ CFU; C57BL/6 WT mice, 5.62 ± 0.20 log_10_ CFU), and the mean bacterial load per spleen was, on average, 2.71 log_10_ CFU greater in *gp91*^−/−^ *phox* mice than in C57BL/6 WT mice (*gp91*^−/−^ *phox* mice, 8.42 ± 0.27 log_10_ CFU; C57BL/6 WT mice, 5.72 ± 0.20 log_10_ CFU).

The input and output pool genomic DNA enriched for transposon junctions was sequenced using the Illumina platform as described in Materials and Methods. A total of 9,356 mutants (4,975 *Mu* and 4,381 Tn*5*) were unambiguously mapped at the level of the single nucleotide to the SL1344 genome (i.e., >94% of the Tn mutants screened were mapped). The reason for the lower number of mapped mutants than the number of mutants in the original input pools could be due to mutants not surviving the initial growth in the input pool and/or the presence of duplicate mutants in the library. A total of 3,139 different genes were disrupted by the 9,356 Tn insertions.

**FIG 1:**
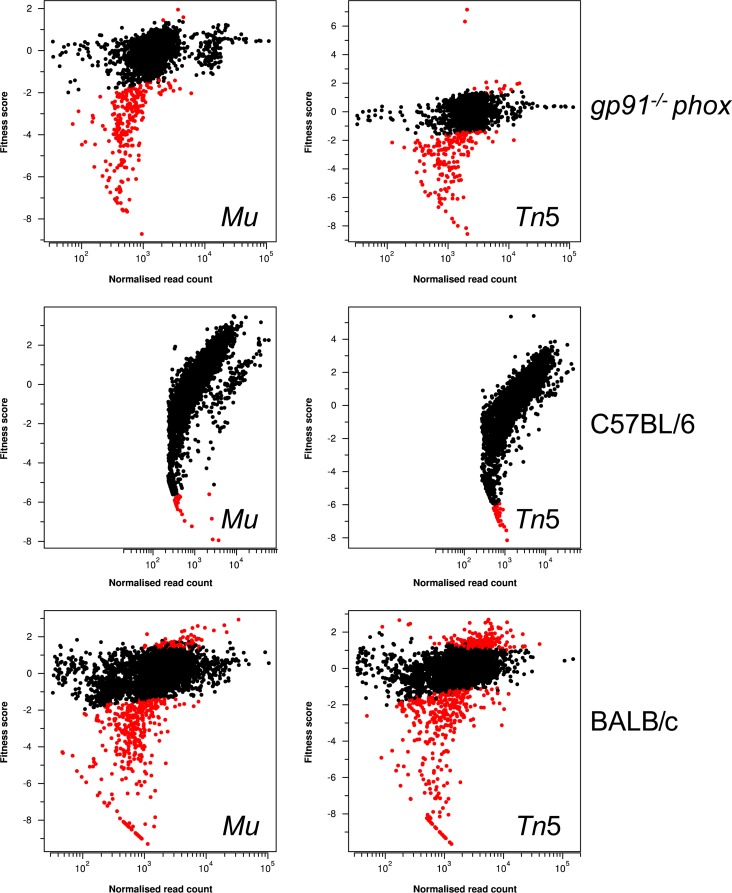
Relative fitness scores (log_2_-fold change) plotted against normalized mean read counts. Fitness scores represent the log_2_-fold change in the sequence read counts of output pools compared with those of the input pools. Output pools were obtained from the livers of *gp91*^−/−^ *phox*, C57BL/6, and BALB/c mice (32). Significantly attenuated mutants, represented as red closed circles, are a population of mutants showing negative fitness scores and an adjusted *P* value (Benjamini-Hochberg) of ≤0.05. Mutants with fitness scores which were not significantly different from zero are shown as black circles. The wide range of fitness scores exhibited in the C57B/L6 mice is indicative of large-scale stochastic loss (i.e., random dropout) of mutants during those infections.

The sites of insertion of all transposons in the *S*. Typhimurium TraDIS mutant libraries, the identities of the genes that are disrupted, and the fitness scores of the mutants are listed in Table S2 in the supplemental material. The fitness scores of the mutants obtained during infection of C57BL/6 mice showed a high proportion of seemingly attenuated mutants (see Fig. S1a in the supplemental material). Additionally, the output read counts from the two replicate C57BL/6 mice did not show a good correspondence (see Fig. S1b), and the fitness scores did not correlate well with those obtained from BALB/c mice (see Fig. S1c) in our previous study (32) (Fig. 1). From these observations, we concluded that the C57BL/6 mice had exhibited stochastic loss of mutants for reasons unrelated to their genotype (i.e., random dropout). In contrast, only a small proportion of mutants were attenuated in the *gp91*^−/−^ *phox* mice, with good correspondence between the replicates and with the data obtained from BALB/c mice (see Fig. S2). The random dropout was not identified in the BALB/c mice, probably because of the increased bacterial load used as the inoculum in this model (∼1 × 10^6^ CFU in the BALB/c mice versus ∼5 × 10^4^ CFU in the C57BL/6 mice), or in the *gp91*^−/−^ *phox* mice due to the absence of bacterial killing in this model. Thus, the attenuated mutants in the C57BL/6 data set are likely to include false negatives due to the random dropout of mutants in this model (i.e., mutants that have been assigned as attenuated but that could be colonization proficient). This prevented an in-depth comparison of the different genes that contributed to fitness in the wild-type C57BL/6 and immunocompromised mice. However, the data from the screen in the *gp91*^−/−^ *phox* mice were robust and provided us with solid foundations to select representative genes from the attenuated mutant lists and test defined allelic replacement mutants of these genes in wild-type and immunocompromised mice.

### Analysis of defined allelic replacement *S*. Typhimurium mutants in C57BL/6 WT and *gp91*^−/−^ *phox* mice.

An ideal live attenuated vaccine would need to colonize but be attenuated in an immunocompetent host and to remain attenuated under conditions where the immune system may be impaired. Candidate genes for the generation of such vaccines will be among those that lead to attenuated mutant phenotypes in spleens and livers of both C57BL/6 WT and *gp91*^−/−^ *phox* mice. We selected 14 genes that fit this pattern, *cydC*, *cydD*, *cysE*, *dksA*, *ftsK*, *miaA*, *nuoK*, *ptsI*, *recD*, *secG*, *seqA*, *sucA*, *thdF*, and *yqiC*, and deleted them by allelic replacement in *S*. Typhimurium. We also generated a double mutant knocking out both *cydC* and *cydD*, a mutant containing a deletion of the entire *suc* operon, a mutant containing a deletion of the entire *nuo* operon, a mutant containing a deletion of the *tol* operon (since many different *tol* mutants were attenuated and *tol* genes previously have been shown to have a role in virulence/fitness in mice [33]), and a mutant containing a deletion of the *tol pal* operon. All of these were tested individually for attenuation in C57BL/6 WT mice compared to levels in the SL1344 wild type, administered by i.v. injection, and at the input doses detailed in Table 2. We also included in the experiment controls mutants lacking *aroC*, *aroC aroD htrA*, and *aroC ssaV*, which have established roles in virulence and have been or are being used as vaccine candidates (34–36). Each gene, or genes, was inactivated separately by λRed recombinase-mediated integration of linear PCR amplicons by homologous recombination. Mice were killed at 7 days p.i. or earlier if clinical signs became apparent (Table 2). Defined mutants of *cydC*, *cydD*, *cydC cydD*, *recD*, *seqA*, and the *suc* operons were not attenuated in the C57BL/6 mice, and mice had to be killed at 3 days p.i., along with the mice infected with wild-type SL1344. Mice infected with defined *ftsK*, *nuoK*, and *nuo* operon mutants were killed on day 4 p.i., as the mice displayed clinical signs. Mice infected with a *dksA* mutant were killed on day 5 p.i. Mice infected with *sucA* and *thdF* mutants displayed clinical signs at 6 days p.i. and were killed, although interestingly viable bacterial counts in the livers and spleens were not high enough to be consistent with the observed signs of infection, indicating that the clinical signs are due to focal infection at other body sites. Mice infected with *cysE*, *miaA*, *ptsI*, *secG*, *tol* operon, *tol pal* operon, and *yqiC* mutants, as well as the controls infected with *aroC*, *aroC aroD htrA*, and *aroC ssaV* mutants, did not display any visible clinical signs on day 7 p.i.

**TABLE 2:** CFU in the organs of C57BL/6 WT mice infected with defined mutants of *S*. Typhimurium, TraDIS fitness scores, and the number of mutants in each gene identified by TraDIS

Mutation	Inoculum (log_10_ CFU)	Organ	Counts in organs (mean log_10_ CFU ± SD)	Day p.i.	TraDIS fitness score
*cydC*	5.60	Liver	8.19 (0.05)	3	−3.3; 1 mutant
		Spleen	8.03 (0.19)		−2.8; 1 mutant
*cydD*	5.51	Liver	8.40 (0.11)	3	−2.6 (upper), −3.8 (lower); 3 mutants
		Spleen	8.34 (0.12)		−2.2 (upper), −4.1 (lower); 3 mutants
*cydC cydD*	5.54	Liver	7.86 (0.28)	3	Not available
		Spleen	8.00 (0.36)		Not available
*recD*	5.70	Liver	8.47 (0.21)	3	−4.9; 1 mutant
		Spleen	8.07 (0.33)		−3.2; 1 mutant
*seqA*	5.65	Liver	8.37 (0.09)	3	−5.5; 1 mutant
		Spleen	8.18 (0.07)		−2.6; 1 mutant
*suc* operon	5.49	Liver	8.54 (0.01)	3	−0.8 (upper), −5.3 (lower); 4 mutants
		Spleen	8.30 (0.04)		−0.3 (upper), −5.9 (lower); 4 mutants
SL1344 WT	3.28	Liver	5.47 (0.23)	3	Not available
		Spleen	5.72 (0.16)		Not available
*ftsK*	5.64	Liver	7.61 (0.21)	4	−5.1; 1 mutant
		Spleen	7.14 (0.22)		−5.8; 1 mutant
*nuoK*	5.72	Liver	8.35 (0.33)	4	−3.3 (upper), −4.8 (lower); 2 mutants
		Spleen	8.05 (0.06)		−2.8 (upper), −4.4 (lower); 2 mutants
*nuo* operon	5.73	Liver	8.35 (0.13)	4	−0.3 (upper), −4.4 (lower); 18 mutants
		Spleen	8.10 (0.13)		−1.7 (upper), −6.6 (lower); 18 mutants
*dksA*	5.69	Liver	8.49	5	−4.3; 1 mutant
		Spleen	8.19		−3.9; 1 mutant
*sucA*	5.71	Liver	5.99 (0.61)	6	−5.4; 1 mutant
		Spleen	6.71 (0.27)		−5.9; 1 mutant
*thdF*	5.48	Liver	7.00 (1.98)	6	−2.4 (upper), −6.4 (lower); 2 mutants
		Spleen	7.07 (1.57)		−2.7 (upper), −3.8 (lower); 2 mutants
*aroC*	5.32	Liver	5.30 (0.16)	7	−4.0 (upper), −4.2 (lower); 2 mutants
		Spleen	5.89 (0.08)		−3.2 (upper), −3.9 (lower); 2 mutants
*aroC aroD htrA*	5.36	Liver	2.63 (0.08)	7	Not available
		Spleen	3.46 (0.11)		Not available
*aroC ssaV*	5.51	Liver	5.02 (0.18)	7	Not available
		Spleen	5.64 (0.05)		Not available
*cysE*	5.51	Liver	5.61 (0.11)	7	−2.8; 1 mutant
		Spleen	5.88 (0.15)		−4.1; 1 mutant
*miaA*	5.53	Liver	4.53 (0.19)	7	−5.0 (upper), −7.2 (lower); 4 mutants
		Spleen	4.68 (0.10)		−4.4 (upper), −5.8 (lower); 4 mutants
*ptsI*	5.57	Liver	6.99 (0.53)	7	−1.5; 1 mutant
		Spleen	6.95 (0.44)		−2.1; 1 mutant
*secG*	5.65	Liver	6.67 (1.09)	7	−5.2; 1 mutant
		Spleen	5.95 (0.48)		−5.7; 1 mutant
*tol* operon	5.74	Liver	3.27 (0.20)	7	−4.4 (upper), −5.4 (lower); 6 mutants
		Spleen	3.93 (0.09)		−4.5 (upper), −9.0 (lower); 6 mutants
*tol pal* operon	5.54	Liver	3.42 (0.10)	7	Not available
		Spleen	4.07 (0.16)		Not available
*yqiC*	5.65	Liver	6.53 (0.72)	7	−6.0; 1 mutant
		Spleen	6.39 (0.21)		−5.9; 1 mutant

**FIG 2:**
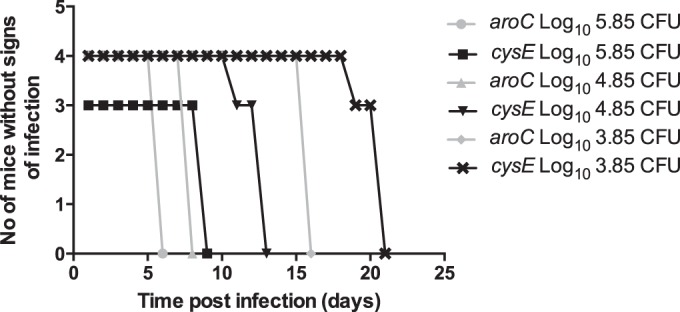
Time to presentation of clinical signs in *gp91*^−/−^ *phox* mice infected with various doses of the SL1344 *cysE* or SL1344 *aroC* mutant. *gp91*^−/−^ *phox* mice were infected i.v. with 5.85 log_10_ CFU, 4.85 log_10_ CFU, or 3.85 log_10_ CFU of either the SL1344 *aroC* (gray symbols) or SL1344 *cysE* (black symbols) mutant, and the time to clinical signs was monitored.

Given their attenuation in WT mice, *cysE*, *miaA*, *ptsI*, *secG*, and *yqiC* mutants, along with the *tol* operon and *tol pal* operon mutants, were used to infect *gp91*^−/−^ *phox* mice. We also included *aroC*, *aroC aroD*, and *ssaV* mutants as controls. Mice were killed at 7 days p.i. or earlier if clinical signs became apparent (Table 3). Mice infected with defined *ptsI*, *ssaV*, *tol* operon, and *tol pal* operon mutants were killed on day 3 p.i., when they displayed clinical signs. Mice infected with *aroC*, *miaA*, and *secG* mutants were killed on day 4 p.i. Mice infected with a *yqiC* mutant were killed on day 5 p.i. when they displayed clinical signs. Mice infected with an *aroC aroD* or *cysE* mutant were killed on day 7 p.i., which was the predefined endpoint of the experiment. The *cysE* and the *aroC aroD* mutants had higher bacterial counts in the organs of the *gp91*^−/−^ *phox* mice than in the C57BL/6 WT mice. In order to determine how long the *gp91*^−/−^ *phox* mice could survive infection with a *cysE* mutant, we performed a small pilot study, infecting *gp91*^−/−^ *phox* mice with 5.72 log_10_ CFU of the SL1344 *cysE* mutant and then allowing the infection to proceed until the mice displayed clinical symptoms. The mice had to be killed on day 8 p.i., when the bacterial loads in the livers were 8.33 ± 0.11 log_10_ CFU and the bacterial loads in the spleens were 7.81 ± 0.05 log_10_ CFU. We next proceeded to compare the *cysE* mutant to an *aroC* mutant in *gp91*^−/−^ *phox* mice (Fig. 2). At three different input doses, the time until the appearance of clinical signs was consistently delayed for the *cysE* mutant compared to that for the *aroC* mutant.

**TABLE 3:** CFU in the organs of *gp91*^−/−^ *phox* mice infected with defined mutants of *S*. Typhimurium, TraDIS fitness scores, and the number of mutants in each gene identified by TraDIS

Mutation	Inoculum (log_10_ CFU)	Organ	Counts in organs (mean log_10_ CFU ± SD)	Day p.i.	TraDIS fitness score
*ptsI*	5.60	Liver	7	3	−4.5; 1 mutant
		Spleen	8.40 (0.06)		−4.0; 1 mutant
*ssaV*	5.69	Liver	9.01 (0.10)	3	−2.9 (upper), −5.3 (lower); 4 mutants
		Spleen	8.63 (0.11)		−3.1 (upper), −5.6 (lower); 4 mutants
*tol* operon	5.48	Liver	8.30 (0.80)	3	−5.8 (upper), −7.6 (lower); 7 mutants
		Spleen	8.00 (1.00)		−5.8 (upper), −6.8 (lower); 7 mutants
*tol pal* operon	5.45	Liver	8.28 (0.29)	3	Not available
		Spleen	7.53 (0.44)		Not available
*aroC*	5.56	Liver	7.83 (0.32)	4	−5.6 (upper), −6.0 (lower); 2 mutants
		Spleen	7.59 (0.48)		−5.3 (upper), −6.5 (lower); 2 mutants
*miaA*	5.60	Liver	9.86 (0.04)	4	−4.4 (upper), −6.1 (lower); 4 mutants
		Spleen	9.27 (0.32)		−5.8 (upper), −7.7 (lower); 4 mutants
*secG*	5.34	Liver	8.85 (0.38)	4	−6.9; 1 mutant
		Spleen	8.64 (0.14)		−7.3; 1 mutant
*yqiC*	5.51	Liver	9.19 (0.21)	5	−0.3; 1 mutant
		Spleen	8.05 (0.13)		−7.7; 1 mutant
*aroC aroD*	5.76	Liver	8.06 (0.14)	7	Not available
		Spleen	7.75 (0.08)		Not available
*cysE*	5.63	Liver	7.89 (0.07)	7	−5.2; 1 mutant
		Spleen	7.55 (0.22)		−5.9; 1 mutant

**FIG 3:**
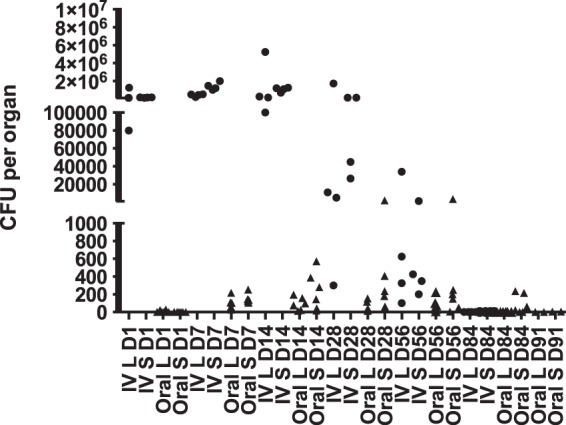
WT C57BL/6 mice were infected i.v. (circles) or orally (triangles) with 5.80 log_10_ CFU or 8.68 log_10_ CFU, respectively, of the SL1344 *cysE* mutant, and bacterial counts in the livers (L) and spleens (S) were monitored at various times postinfection until the bacteria had cleared from the organs.

As a prerequisite to a vaccination study, we determined the time it took for the SL1344 *cysE* mutant to be cleared from the liver and spleen of WT C57BL/6 mice (Fig. 3). We found that by day 84 p.i., mice infected i.v. had cleared all of the SL1344 *cysE* mutant from the liver and spleen, while in the orally infected group it took until day 91 p.i. for the organs to be clear of the SL1344 *cysE* mutant.

**FIG 4:**
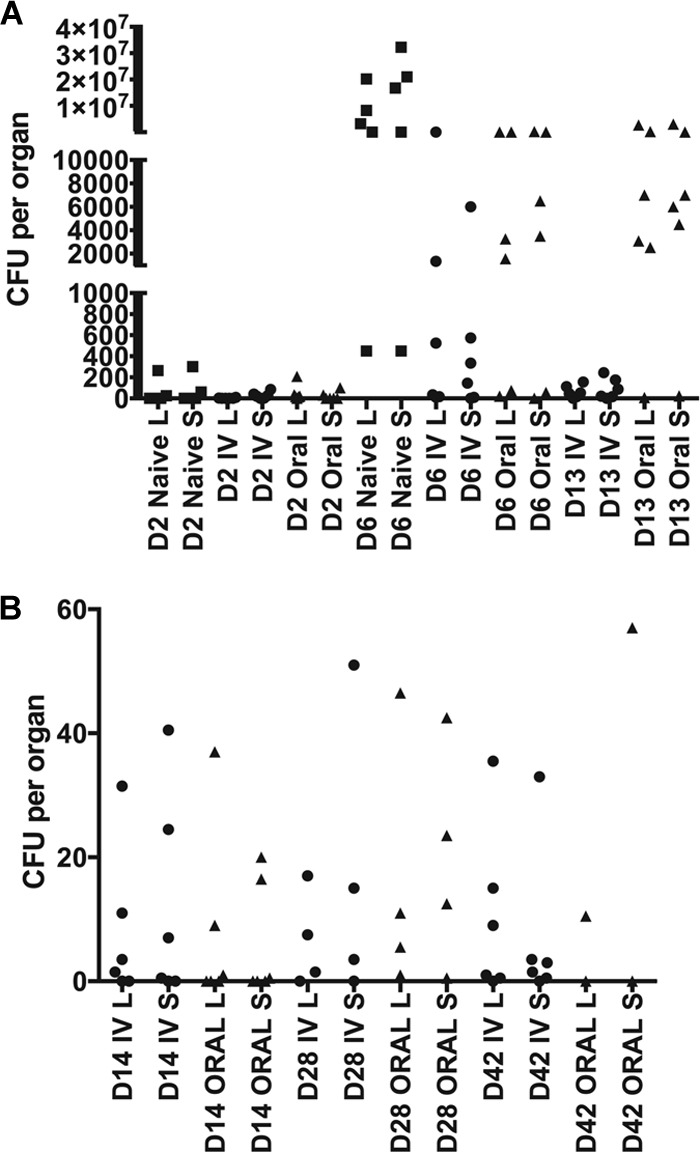
Vaccination study, immunizing C57BL/6 WT mice with the SL1344 *cysE* mutant, and subsequent challenge with WT SL1344. (A) WT C57BL/6 mice were vaccinated with the SL1344 *cysE* mutant administered at either 5.7 log_10_ CFU i.v. or 8.5 log_10_ CFU orally. At 91 days postimmunization, the vaccinated mice and an unvaccinated group of mice (naive control) were challenged orally with 8.8 log_10_ CFU of virulent SL1344. Bacterial counts in the livers (L) and spleens (S) were monitored at various times postchallenge (note that one mouse from the orally vaccinated group had to be killed on day 9 postchallenge, since it was displaying signs of infection). (B) WT C57BL/6 mice were vaccinated with the SL1344 *cysE* mutant administered at either 5.8 log_10_ CFU i.v. or 8.7 log_10_ CFU orally. At 91 days postimmunization, the vaccinated mice were challenged orally with 8.8 log_10_ CFU or virulent SL1344. Bacterial counts in the livers and spleens were monitored at various times postchallenge (note that one mouse from both the orally vaccinated and the i.v. vaccinated group had to be killed on day 9 postchallenge and one mouse each from the orally vaccinated group had to be killed on day 18, day 19, and day 34 postchallenge, since they were displaying signs of infection).

We next vaccinated WT C57BL/6 mice, either i.v. or orally, with the SL1344 *cysE* mutant and then challenged the mice, 91 days postimmunization, orally with virulent SL1344 and monitored the bacterial counts in the livers and spleens at various times postinfection (Fig. 4). The majority of the vaccinated mice were protected from challenge with the virulent SL1344. However, during the challenge experiment it was necessary to kill six mice (five from the orally immunized group and one from the i.v. immunized group) because they were displaying signs of infection. Thus, 97% of the SL1344 *cysE* mutant i.v. vaccinated C57BL/6 mice and 86% of the SL1344 *cysE* mutant orally vaccinated C57BL/6 mice were protected against challenge with virulent SL1344.

### Conclusion.

Here, we have presented a report of the fitness of random Tn mutants of *S*. Typhimurium *in vivo* and described attenuated mutants based on the TraDIS analysis of pools of Tn mutants screened in C57BL/6 WT and *gp91*^−/−^ *phox* mice. Our study provides proof-of-principle data on the feasibility of using TraDIS analysis in combination with infections of gene-targeted animals to allow the identification of bacterial mutants as live vaccine candidates that would be safe in situations where the immune system is impaired.

We show that through TraDIS analysis it is possible to identify novel mutations in *S*. Typhimurium genes that still allow colonization in WT mice but importantly also reduce bacterial growth rate *in vivo* in severely immunodeficient *gp91*^−/−^ *phox* mice. It is worth noting that the variance of defined mutants from their TraDIS fitness scores (Tables 2 and 3) likely is due to differences in competition dynamics for a given mutant relative to those of coscreened mutant bacteria, as we previously demonstrated (32), and the differences in time points at which the bacteria were enumerated p.i. between the TraDIS screen and the defined mutant experiments. Thus, some of the attenuated mutants identified in the screen are virulent when tested individually, while some others retain the attenuation; therefore, the screen is useful but requires further evaluation of mutations individually.

Through the TraDIS screens and validation of defined mutants, we identified that the SL1344 *cysE* mutant was attenuated for growth, was cleared from WT C57BL/6 mice, and had reduced growth in *gp91*^−/−^ *phox* mice, although these mice succumbed to the infection 8 days p.i. The SL1344 *cysE* mutant offered protection when given i.v. or orally as a live vaccine to C57BL/6 WT mice; however, a few of the vaccinated mice succumbed to the challenge with the virulent SL1344, indicating that *cysE* does not offer full protection to all vaccinated animals.

Our work did not identify a single-gene *S*. Typhimurium mutant that would colonize WT animals at levels sufficient to be predicted to be immunogenic and that also would be completely unable to cause disease in the severely immunodeficient animals used in this study. The attractive possibility that different combinations of mutations could yield this ideal vaccine candidate remains to be explored. However, even mutations that reduce growth in immunocompromised hosts would be advantageous and could be used to construct live strains in a way that would crucially enable more time for medical intervention should an immunocompromised vaccinee develop signs of infection. Furthermore, the animal model that we used was of extreme immunodeficiency, while it is likely that in the field one would encounter situations where macrophage function is only partially impaired. Therefore, it remains to be established whether some of the mutants identified from this study would be safer in models of partial immunodeficiency.

Extending the approach reported here to other gene-targeted mouse strains has the potential in the future to generate a more comprehensive picture of the functional interactions between the bacterial and host genomes and to identify bacterial mutants as live vaccine candidates that would be safe across a wide range of immunodeficiencies.

## Supplementary Material

Supplemental material
