# Supplementary material for: Genes Required for the Fitness of Salmonella enterica Serovar Typhimurium during Infection of Immunodeficient gp91−/− phox Mice
Source: Infect Immun. 2016 Mar 24;84(4):989–97. doi: 10.1128/IAI.01423-15 (PMC4807482; doi:10.1128/IAI.01423-15)
Supplement: Supplemental material [file supp_84_4_989__index.html]

Supplemental material 

# Genes Required for the fitness of *Salmonella enterica* serovar Typhimurium During Infection of Immunodeficient *gp91-/-phox* Mice

## Supplemental material

- Supplemental file 1 -

  Supplemental methods. Fig. S1. Fitness scores obtained during infection of C57/BL6 versus BALB/c mice, and comparison of raw number of reads obtained for each mutant from the livers of two replicate C57/BL6 mice. Fig. S2. Fitness scores obtained during infection of *gp91*−/−*phox* versus BALB/c mice, and comparison of raw number of reads obtained for each mutant from the livers of two replicate *gp91*−/−*phox* mice. Table S1. Primer sequences used in this study.

  PDF, 827K
- Supplemental file 2 -

  Table S2. Raw read counts, fitness scores, and adjusted *P* values for 9,356 transposon mutants during infection of *gp91*−/−*phox*, C57/BL6, and BALB/c mice.

  XLSX, 2.3M
- Supplemental file 3 -

  Table S3. Raw read counts, fitness scores, and adjusted *P* values for 447 transposon mutants significantly attenuated during infection of *gp91*−/−*phox* mice.

  XLSX, 126K
