# Supplementary material for: Genes Required for the Fitness of Salmonella enterica Serovar Typhimurium during Infection of Immunodeficient gp91−/− phox Mice
Source: Infect Immun. 2016 Mar 24;84(4):989–97. doi: 10.1128/IAI.01423-15 (PMC4807482; doi:10.1128/IAI.01423-15)
Supplement: Supplemental material [file IAI.01423-15_zii999091634so1.pdf]

Genes Required for the fitness of *Salmonella enterica* serovar Typhimurium During Infection of Immunodeficient *gp91$^{-/-}$phox* Mice

Running title: *Salmonella* TraDIS in immunodeficient mice

Andrew J. Grant[1#], Olusegun Oshota[1], Roy R. Chaudhuri[1a], Matthew Mayho[2], Sarah E. Peters[1], Simon Clare[2], Duncan J. Maskell[1], Pietro Mastroeni[1]

1 Department of Veterinary Medicine, University of Cambridge, Madingley Road, Cambridge, UK.

2 Wellcome Trust Sanger Institute, Wellcome Trust Genome Campus, Hinxton, Cambridge, UK.

a Current address: Department of Molecular Biology and Biotechnology, University of Sheffield, Firth Court, Western Bank, Sheffield, UK.

[#] To whom correspondence should be addressed: ajg60@cam.ac.uk

SUPPLEMENTAL INFORMATION

MATERIALS AND METHODS

Details of *S.* Typhimurium defined mutant generation.

Generation of SL1344 *aroC* mutant. PCR was used to amplify the chloramphenicol resistance cassette from pACYC184 (S1) with 5' and 3' 60 bp homology arms complementary to the flanking regions of *aroC* using the primers ajg806 and ajg807 (Table S2). The resulting product was further amplified using the primers ajg805 and ajg808 (Table S2) to generate sufficient product for chromosomal integration into SL1344. The correct genomic rearrangement in the resultant mutant was confirmed by PCR and sequencing (data not shown) using the primers ajg804 and ajg809 (Table S2).

Generation of S12023 *aroC/aroD* mutant. PCR was used to amplify the kanamycin resistance cassette from pACYC177 (S1) with 5' and 3' 60 bp homology arms complementary to the flanking regions of *aroD* using the primers ajg812 and ajg813 (Table S2). The resulting product was further amplified using the primers ajg811 and ajg814 (Table S2) to generate sufficient product for chromosomal integration into SL1344 *aroC*. The correct genomic rearrangement in the resultant mutant was confirmed by PCR and sequencing (data not shown) using the primers ajg810 and ajg815 (Table S2).

Generation of S12023 *aroC/aroD/htrA* mutant. PCR was used to amplify the kanamycin resistance cassette from pACYC177 (S1) with 5' and 3' 60 bp

homology arms complementary to the flanking regions of *aroD* using the primers ajg812 and ajg813 (Table S2). The resulting product was further amplified using the primers ajg811 and ajg814 (Table S2) to generate sufficient product for chromosomal integration into SL1344 *aroC*. The correct genomic rearrangement in the resultant mutant was confirmed by PCR and sequencing (data not shown) using the primers ajg810 and ajg815 (Table S2). Subsequently, PCR was used to amplify the tetracycline resistance cassette from pBR322 (S2) with 5' and 3' 60 bp homology arms complementary to the flanking regions of *htrA* using the primers ajg818 and ajg819 (Table S2). The resulting product was further amplified using the primers ajg817 and ajg820 (Table S2) to generate sufficient product for chromosomal integration into SL1344 *aroC/aroD*. The correct genomic rearrangement in the resultant mutant was confirmed by PCR and sequencing (data not shown) using the primers ajg816 and ajg821 (Table S2).

Generation of SL1344 *aroC/ssaV* mutant. PCR was used to amplify the kanamycin resistance cassette from pACYC177 (S1) with 5' and 3' 60 bp homology arms complementary to the flanking regions of *ssaV* and including the first 16 nucleotides and last 11 nucleotides of *ssaV*, using the primers ajg824 and ajg825 (Table S2). The resulting product was further amplified using the primers ajg823 and ajg826 (Table S2) to generate sufficient product for chromosomal integration into SL1344 *aroC*. The correct genomic rearrangement in the resultant mutant was confirmed by PCR and sequencing (data not shown) using the primers ajg822 and ajg827 (Table S2).

Generation of SL1344 *cydC* mutant. PCR was used to amplify the chloramphenicol resistance cassette from pACYC184 (S1) with 5' and 3' 60 bp homology arms complementary to the flanking regions of *cydC* using the primers ajg836 and ajg837 (Table S2). The resulting product was further amplified using the primers ajg835 and ajg838 (Table S2) to generate sufficient product for chromosomal integration into SL1344. The correct genomic rearrangement in the resultant mutant was confirmed by PCR and sequencing (data not shown) using the primers ajg834 and ajg839 (Table S2).

Generation of SL1344 *cydC/cydD* mutant. PCR was used to amplify the chloramphenicol resistance cassette from pACYC184 (S1) with 5' and 3' 60 bp homology arms complementary to the flanking regions of *cydC* cydD using the primers ajg842 and ajg843 (Table S2). The resulting product was further amplified using the primers ajg841 and ajg844 (Table S2) to generate sufficient product for chromosomal integration into SL1344. The correct genomic rearrangement in the resultant mutant was confirmed by PCR and sequencing (data not shown) using the primers ajg840 and ajg845 (Table S2).

Generation of SL1344 *cydD* mutant. PCR was used to amplify the chloramphenicol resistance cassette from pACYC184 (S1) with 5' and 3' 60 bp homology arms complementary to the flanking regions of *cydD* using the primers ajg848 and ajg849 (Table S2). The resulting product was further amplified using the primers ajg847 and ajg850 (Table S2) to generate sufficient product for chromosomal integration into SL1344. The correct genomic rearrangement in the

resultant mutant was confirmed by PCR and sequencing (data not shown) using the primers ajg846 and ajg851 (Table S2).

Generation of SL1344 *cysE* mutant. PCR was used to amplify the chloramphenicol resistance cassette from pACYC184 (S1) with 5' and 3' 60 bp homology arms complementary to the flanking regions of *cysE* using the primers ajg854 and ajg855 (Table S2). The resulting product was further amplified using the primers ajg853 and ajg856 (Table S2) to generate sufficient product for chromosomal integration into SL1344. The correct genomic rearrangement in the resultant mutant was confirmed by PCR and sequencing (data not shown) using the primers ajg852 and ajg857 (Table S2).

Generation of SL1344 *dksA* mutant. PCR was used to amplify the chloramphenicol resistance cassette from pACYC184 (S1) with 5' and 3' 60 bp homology arms complementary to the flanking regions of *dksA* using the primers ajg860 and ajg861 (Table S2). The resulting product was further amplified using the primers ajg859 and ajg862 (Table S2) to generate sufficient product for chromosomal integration into SL1344. The correct genomic rearrangement in the resultant mutant was confirmed by PCR and sequencing (data not shown) using the primers ajg858 and ajg863 (Table S2).

Generation of SL1344 *ftsK* mutant. PCR was used to amplify the chloramphenicol resistance cassette from pACYC184 (S1) with 5' and 3' 60 bp

homology arms complementary to the flanking regions of *ftsK* using the primers ajg866 and ajg867 (Table S2). The resulting product was further amplified using the primers ajg865 and ajg868 (Table S2) to generate sufficient product for chromosomal integration into SL1344. The correct genomic rearrangement in the resultant mutant was confirmed by PCR and sequencing (data not shown) using the primers ajg864 and ajg869 (Table S2).

Generation of SL1344 *miaA* mutant. PCR was used to amplify the chloramphenicol resistance cassette from pACYC184 (S1) with 5' and 3' 60 bp homology arms complementary to the flanking regions of *miaA* and including the first 8 nucleotides of *miaA* using the primers ajg896 and ajg897 (Table S2). The resulting product was further amplified using the primers ajg895 and ajg898 (Table S2) to generate sufficient product for chromosomal integration into SL1344. The correct genomic rearrangement in the resultant mutant was confirmed by PCR and sequencing (data not shown) using the primers ajg894 and ajg899 (Table S2).

Generation of SL1344 *nuo* operon mutant. PCR was used to amplify the chloramphenicol resistance cassette from pACYC184 (S1) with 5' and 3' 60 bp homology arms complementary to the flanking regions of the *nuo* operon using the primers ajg902 and ajg903 (Table S2). The resulting product was further amplified using the primers ajg901 and ajg904 (Table S2) to generate sufficient product for chromosomal integration into SL1344. The correct genomic rearrangement in the resultant mutant was confirmed by PCR and sequencing (data not shown) using the primers ajg900 and ajg905 (Table S2).

Generation of SL1344 *nuoK* mutant. PCR was used to amplify the chloramphenicol resistance cassette from pACYC184 (S1) with 5' and 3' 60 bp homology arms complementary to the flanking regions of the *nuo* operon using the primers ajg908 and ajg909 (Table S2). The resulting product was further amplified using the primers ajg907 and ajg910 (Table S2) to generate sufficient product for chromosomal integration into SL1344. The correct genomic rearrangement in the resultant mutant was confirmed by PCR and sequencing (data not shown) using the primers ajg906 and ajg911 (Table S2).

Generation of SL1344 *ptsI* mutant. PCR was used to amplify the chloramphenicol resistance cassette from pACYC184 (S1) with 5' and 3' 60 bp homology arms complementary to the flanking regions of *ptsI* using the primers ajg932 and ajg933 (Table S2). The resulting product was further amplified using the primers ajg931 and ajg934 (Table S2) to generate sufficient product for chromosomal integration into SL1344. The correct genomic rearrangement in the resultant mutant was confirmed by PCR and sequencing (data not shown) using the primers ajg930 and ajg935 (Table S2).

Generation of SL1344 *recD* mutant. PCR was used to amplify the chloramphenicol resistance cassette from pACYC184 (S1) with 5' and 3' 60 bp homology arms complementary to the flanking regions of *recD* and including the first 4 nucleotides of *recD* using the primers ajg950 and ajg51 (Table S2). The resulting

product was further amplified using the primers ajg949 and ajg952 (Table S2) to generate sufficient product for chromosomal integration into SL1344. The correct genomic rearrangement in the resultant mutant was confirmed by PCR and sequencing (data not shown) using the primers ajg948 and ajg953 (Table S2).

Generation of SL1344 *secG* mutant. PCR was used to amplify the chloramphenicol resistance cassette from pACYC184 (S1) with 5' and 3' 60 bp homology arms complementary to the flanking regions of *secG* using the primers ajg992 and ajg993 (Table S2). The resulting product was further amplified using the primers ajg991 and ajg994 (Table S2) to generate sufficient product for chromosomal integration into SL1344. The correct genomic rearrangement in the resultant mutant was confirmed by PCR and sequencing (data not shown) using the primers ajg990 and ajg995 (Table S2).

Generation of SL1344 *seqA* mutant. PCR was used to amplify the chloramphenicol resistance cassette from pACYC184 (S1) with 5' and 3' 60 bp homology arms complementary to the flanking regions of *seqA* using the primers ajg998 and ajg999 (Table S2). The resulting product was further amplified using the primers ajg997 and ajg1000 (Table S2) to generate sufficient product for chromosomal integration into SL1344. The correct genomic rearrangement in the resultant mutant was confirmed by PCR and sequencing (data not shown) using the primers ajg996 and ajg1001 (Table S2).

Generation of SL1344 *sucA* mutant. PCR was used to amplify the chloramphenicol resistance cassette from pACYC184 (S1) with 5' and 3' 60 bp homology arms complementary to the flanking regions of *sucA* using the primers ajg1076 and ajg1077 (Table S2). The resulting product was further amplified using the primers ajg1075 and ajg1078 (Table S2) to generate sufficient product for chromosomal integration into SL1344. The correct genomic rearrangement in the resultant mutant was confirmed by PCR and sequencing (data not shown) using the primers ajg1074 and ajg1079 (Table S2).

Generation of SL1344 *suc* operon mutant. PCR was used to amplify the chloramphenicol resistance cassette from pACYC184 (S1) with 5' and 3' 60 bp homology arms complementary to the flanking regions of the *suc* operon using the primers ajg1082 and ajg1083 (Table S2). The resulting product was further amplified using the primers ajg1081 and ajg1084 (Table S2) to generate sufficient product for chromosomal integration into SL1344. The correct genomic rearrangement in the resultant mutant was confirmed by PCR and sequencing (data not shown) using the primers ajg1080 and ajg1085 (Table S2).

Generation of SL1344 *thdF* mutant. PCR was used to amplify the chloramphenicol resistance cassette from pACYC184 (S1) with 5' and 3' 60 bp homology arms complementary to the flanking regions of *thdF* using the primers ajg1052 and ajg1053 (Table S2). The resulting product was further amplified using the primers ajg1051 and ajg1054 (Table S2) to generate sufficient product for chromosomal integration into SL1344. The correct genomic rearrangement in the

resultant mutant was confirmed by PCR and sequencing (data not shown) using the primers ajg1050 and ajg1055 (Table S2).

Generation of SL1344 *tol* operon mutant. PCR was used to amplify the chloramphenicol resistance cassette from pACYC184 (S1) with 5' and 3' 60 bp homology arms complementary to the flanking regions of the *tol* operon and including the last 4 nucleotides of *tolQ* using the primers ajg1058 and ajg1059 (Table S2). The resulting product was further amplified using the primers ajg1057 and ajg1060 (Table S2) to generate sufficient product for chromosomal integration into SL1344. The correct genomic rearrangement in the resultant mutant was confirmed by PCR and sequencing (data not shown) using the primers ajg1056 and ajg1061 (Table S2).

Generation of SL1344 *tol/pal* operon mutant. PCR was used to amplify the chloramphenicol resistance cassette from pACYC184 (S1) with 5' and 3' 60 bp homology arms complementary to the flanking regions of the *tol pal* operon and including the last 4 nucleotides of *tolQ* using the primers ajg1064 and ajg1065 (Table S2). The resulting product was further amplified using the primers ajg1063 and ajg1066 (Table S2) to generate sufficient product for chromosomal integration into SL1344. The correct genomic rearrangement in the resultant mutant was confirmed by PCR and sequencing (data not shown) using the primers ajg1062 and ajg1067 (Table S2).

Generation of SL1344 *yqiC* mutant. PCR was used to amplify the chloramphenicol resistance cassette from pACYC184 (S1) with 5' and 3' 60 bp homology arms complementary to the flanking regions of *yqiC* using the primers ajg1070 and ajg1071 (Table S2). The resulting product was further amplified using the primers ajg1069 and ajg1072 (Table S2) to generate sufficient product for chromosomal integration into SL1344. The correct genomic rearrangement in the resultant mutant was confirmed by PCR and sequencing (data not shown) using the primers ajg1068 and ajg1073 (Table S2).

SUPPLEMENTAL REFERENCES

S2 **Chang, C.Y. and Cohen, S.N.** 1978. Construction and characterization of amplifiable multicopy DNA cloning vehicles derived from the P15A cryptic miniplasmid. J. Bacteriol. **134**: 1141-1156.

S2 **Bolivar, F. Rodriguez, R.L. Greene, P.J. Betlach, M.C. Heyneker, H.L. and Boyer, H.W.** 1977. Construction and characterization of new cloning vehicles. II. A multipurpose cloning system. *Gene*. **2**: 95-113.

S3 **Chaudhuri, R.R. Morgan, E. Peters, S.E. Pleasance, S.J. Hudson, D.L. Davies, H.M. Wang, J. van Diemen, P.M. Buckley, A.M. Bowen, A.J. Pullinger, G.D. Turner, D.J. Langridge, G.C. Turner, A.K. Parkhill, J. Charles, I.G. Maskell, D.J. and Stevens, M.P.** 2013. Comprehensive assignment of roles for Salmonella typhimurium genes in intestinal colonization of food-producing animals. PLoS. Genet. **9**(4): e1003456.

FIGURES AND TABLE LEGENDS

Figure S1. (A) Histogram of fitness scores obtained during infection of C57/BL6 mice. (B) Fitness scores obtained for mutants during infection of C57/BL6 mice compared with those obtained during infection of BALB/c mice (S3). (C) Comparison of raw number of reads obtained for each mutant from the livers of two replicate C57/BL6 mice.

Figure S2. (A) Histogram of fitness scores obtained during infection of *gp91*$^{-/-}$*phox* mice. (B) Fitness scores obtained for mutants during infection of *gp91*$^{-/-}$*phox* mice compared with those obtained during infection of BALB/c mice (S3). (C) Comparison of raw number of reads obtained for each mutant from the livers of two replicate *gp91*$^{-/-}$*phox* mice mice.

Table S1. Primer sequences used in this study.

Table S2. Raw read counts, fitness scores and adjusted P values for 9,356 transposon mutants during infection of *gp91*$^{-/-}$*phox*, C57/BL6 and BALB/c mice.

Table S3. Raw read counts, fitness scores and adjusted P values for 447 transposon mutants significantly attenuated during infection of *gp91*$^{-/-}$*phox* mice.

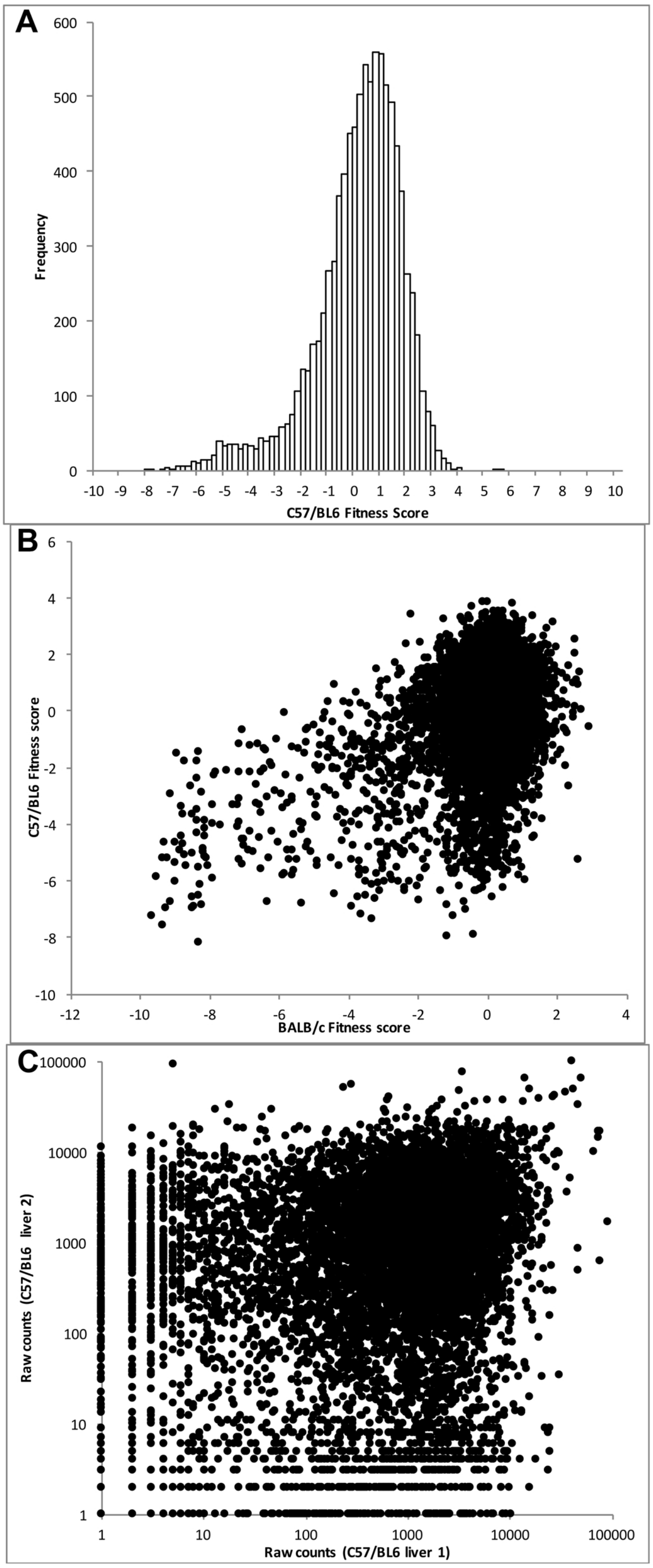

Figure S1. (A) Histogram of fitness scores obtained during infection of C57/BL6 mice. (B) Fitness scores obtained for mutants during infection of C57/BL6 mice

compared with those obtained during infection of BALB/c mice (S3). (C) Comparison of raw number of reads obtained for each mutant from the livers of two replicate C57/BL6 mice.

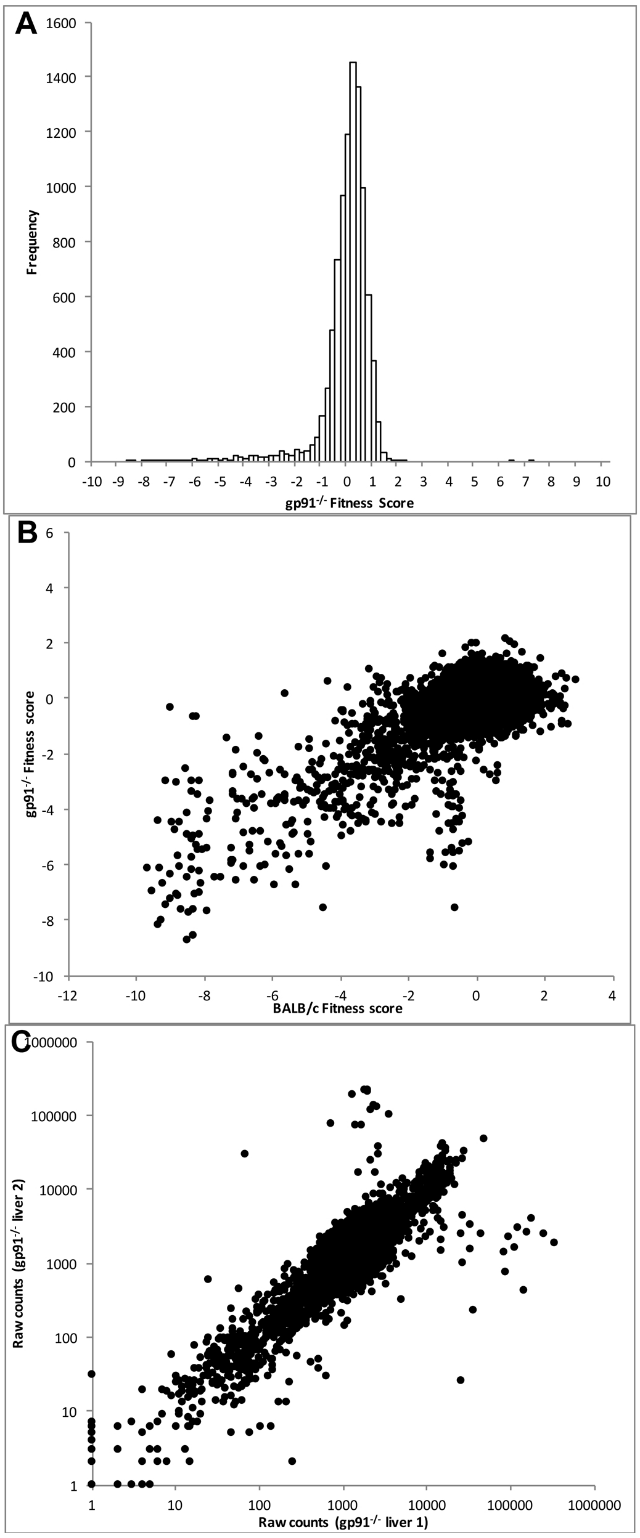

Figure S2. (A) Histogram of fitness scores obtained during infection of *gp91$^{-/-}$phox* mice. (B) Fitness scores obtained for mutants during infection of *gp91$^{-/-}$phox* mice

compared with those obtained during infection of BALB/c mice (S3). (C) Comparison of raw number of reads obtained for each mutant from the livers of two replicate *gp91*$^{-/-}$*phox* mice mice.

Primer	Sequence 5’ to 3’
SplA5_top	gagatcggtctcggcattcctgctgaaccgctcttccgatct
SplA5_bottom	/5phos/gatcggaagagcggttcagcaggtttttttttcaaaaaaa
Mu_AG_5’PCR	aatgatacggcgaccaccgagatctacaccgaattcattaccctgttatccctatttaggtgac
Mu_AG_3’PCR	aatgatacggcgaccaccgagatctacacggatcctctagagtcgactggcaaac
Tn5_AG_5’PCR	aatgatacggcgaccaccgagatctacacctaccctgtggaacacctacatctgtattaacg
Tn5_AG_3’PCR	aatgatacggcgaccaccgagatctacacggatcctctagagtcgactggcaaac
Mu_AG_5’seq	gtgaaacgctttcgcgtttttcgtgcg
Mu_AG_3’seq	gtgaaacgctttcgcgtttttcgtgcg
Tn5_AG_5’seq	ccctgttatccctatttaggtgacactatagaagagatgtgta
Tn5_AG_3’seq	atgggtattatgggtaatacgactcactatagggagatgtgta
SplAP5.1	caagcagaagacggcatacgagataacgtgatgagatcggtctcggcattcc
SplAP5.2	caagcagaagacggcatacgagataaacatcggagatcggtctcggcattcc
ajg804	ccgttcacctggctggagttt
ajg805	acatttcaatatttataaaga
ajg806	acatttcaatatttataaagattaaacacgcaaacgacaacaacgataacggagccgtgcgacgcactttgcgccgaat
ajg807	cgccaggctggcgctactgacaaaccatgccagcagcgcaatcgcggtttttttcatttcttacgccccgccctgccact
ajg808	cgccaggctggcgctactgac
ajg809	atgatgcatccgttggcaaag
ajg810	atgttgttatggcaaggagcc
ajg811	gggattcacagcctgaccggt
ajg812	gggattcacagcctgaccggtaaaattataattgcgacgaatgacaatgaagggtaccaactcaaaatctctgatgttac
ajg813	gtcagataactatttattttattgtggcagaaagagaatattccgccacacgataaagtattagaaaaactcatcgagca
ajg814	gtcagataactatttatttta
ajg815	ttatccctgaaaacaatatcg
ajg816	tcgaacagtaaatggactttt
ajg817	ataaaatgaatctgacgtaca
ajg818	ataaaatgaatctgacgtacacagcaattttgcgttacctgttaatcgagattgaaacacttgacagcttatcatcgata
ajg819	tgtgggggtttcacagaaaagtgttgccccttccatggcggaagggggacaaaggtgatcaggtcgaggtggcccggc
ajg820	tgtgggggtttcacagaaaa
ajg821	tgagcagagcacaagcataag
ajg822	gtattcctcaacgattatttt
ajg823	tggttacgattacatcatcga
ajg824	tggttacgattacatcatcgacaaataaaatttctggagtcgcaatgcgttcatggttagctcaaaatctctgatgttac
ajg825	acaataaccatcgggggcggatatttcagcctcagacgttgcatcaattcattcttcatttagaaaaactcatcgagca
ajg826	acaataaccatcgggggcgg
ajg827	cagcacaactcgcccataaat
ajg834	agttggaagatctcgccgact
ajg835	ctcagcgccgccaacggcgct
ajg836	ctcagcgccgccaacggcgcttttgcgacgttattggctcaccgtcaggaggatatttaacgacgcactttgcgccgaat
ajg837	gctgaaccagacgcatgacggcgaaactccagtacgcaagtcggatcgttcaataatagcttacgccccgccctgccact
ajg838	gctgaaccagacgcatgacgg
ajg839	gctgaaccagacgcatgacgg
ajg840	gatggtctggccgacgcgagc
ajg841	tttgcgtcgttgtaacattgc
ajg842	tttgcgtcgttgtaacattgccctgcctgaaattccaataactcacctgctaagcgtgcacgacgcactttgcgccgaat
ajg843	gctgaaccagacgcatgacggcgaaactccagtacgcaagtcggatcgttcaataatagcttacgccccgccctgccact
ajg844	gctgaaccagacgcatgacgg
ajg845	atcgccgcccagcgccagtaa
ajg846	gatggtctggccgacgcgagc
ajg847	tttgcgtcgttgtaacattgc
ajg848	tttgcgtcgttgtaacattgccctgcctgaaattccaataactcacctgctaagcgtgcacgacgcactttgcgccgaat
ajg849	cagcgttagcatccatttatggcgtttataaagcgtcagataagggagtagcgcgcatttacgccccgccctgccact
ajg850	cagcgttagcatccatttatg
ajg851	gcggacgcggacaggaaccag
ajg852	gcgcgcgaggcagcattaacg
ajg853	cataaacgacccaacccgcac
ajg854	cataaacgacccaacccgcacagaacgggttggtcgttttctgcccgtctggagtaagcccgacgcactttgcgccgaat

ajg855	gctccgctgttccgggattgcactccatcggaacagcgtttttagttgtaccgcgcaatttacgccccgccctgccact
ajg856	gctccgctgttccgggattgc
ajg857	atatctgtttatcgtcggccc
ajg858	gtgcgtaaatacgcttttcct
ajg859	atagcgacctgattttccccc
ajg860	atagcgacctgattttcccccgaacatggggatcgatagtgcgtgttaaggagaagcaaccgacgcactttgcgccgaat
ajg861	caataaagaataaggcgggaaaactcccgcctgtcataaatagggtagaaacgaacgggattacgccccgccctgccact
ajg862	caataaagaataaggcgggaa
ajg863	gattaacgagccgaaatgcag
ajg864	cgctaacacggaacaggtgca
ajg865	ccggtgctgttgtccgtttta
ajg866	ccggtgctgttgtccgttttagcatcgggcgggaaaagcctggaacctggagagccttttcgacgcactttgcgccgaat
ajg867	agaaaatactgaattttgttgccggatggcagcctgcacagcgccatccggcttacaggtttacgccccgccctgccact
ajg868	agaaaatactgaattttgttg
ajg869	ttattcctcaggtttcgacac
ajg894	cgcagtggtcgatggcgcagg
ajg895	ggtggcctgttacaacctgtt
ajg896	ggtggcctgttacaacctgttgatttacattcggcgatgaacgccctgaagcatgaatgacgacgcactttgcgccgaat
ajg897	ttgtactttgaacctttcgattctgaaaaattgcgcacgatacgtctcaattgtacacatttacgccccgccctgccact
ajg898	ttgtactttgaacctttcgat
ajg899	cacgttcccgacgcaatgcgt
ajg900	gtaaaaaaccacatcaattag
ajg901	atgtggcgcccatctgccgta
ajg902	atgtggcgcccatctgccgtaaagagcagagaaactggcgctacttttgatgagtaagcacgacgcactttgcgccgaat
ajg903	tattgctcatcagcctcaaccgccgataaatcggcggttattgacatcatcaacgcggcattacgccccgccctgccact
ajg904	tattgctcatcagcctcaacc
ajg905	gcctcacccaacgagtctatg
ajg906	tggcggtcgaactggcgtcta
ajg907	gaagtgctaagcaaccgcgcc
ajg908	gaagtgctaagcaaccgcgccgatgaccgcgcgaaaagaaaaacggaggagcgcgcatgacgacgcactttgcgccgaat
ajg909	gaacgccagcagtacaaagccaatcaatggcagaataatggttaaggcaagcatgttcatttacgccccgccctgccact
ajg910	gaacgccagcagtacaaagcc
ajg911	aacgctgtcaccaacgccgcc
ajg930	ctcaaggcaccgtcgtcacca
ajg931	gaactcgagtaagtttttttc
ajg932	gaactcgagtaagtttttttccgggttcttttaaaaatcagtcacaagtaaggtagggttcgacgcactttgcgccgaat
ajg933	agtttatcgaacaaacccatgatcttctcctaagcagtaaattgggccgcatctcgtggattacgccccgccctgccact
ajg934	agtttatcgaacaaacccatg
ajg935	tgttgacgatctcgccagaga
ajg948	gcggcgtcatctatctctttt
ajg949	gcgttaattaaccaactggat
ajg950	gcgttaattaaccaactggatgatatgtttgcaggtgaaatgagtgaggaggcgcaatgacgacgcactttgcgccgaat
ajg951	gaaagcatcatttggaggccggataagacgctacgcgtcgccatccggcgagaactcgtcttacgccccgccctgccact
ajg952	gaaagcatcatttggaggccg
ajg953	agcgacgttcaaaggtgctga
ajg990	atacaatgcgcttaaattgcc
ajg991	aatcccgctttattggtagaa
ajg992	aatcccgctttattggtagaagcattggtacgcggcaactccgcaaggaacaggttgattcgacgcactttgcgccgaat
ajg993	gcactaccacctcaaggtagcgtgtctaccaattccaccacctcggcacggatactgctattacgccccgccctgccact
ajg994	gcactaccacctcaaggtagc
ajg995	gaaaaaaaagacgcttttcag
ajg996	gaccattctgccactgggctg
ajg997	gggatttgttcctataatccc
ajg998	gggatttgttcctataatcccaatgacttgtattcagcaaagacatcgcactggattaagcgacgcactttgcgccgaat
ajg999	ttgcgcgggttgtcctgcgcggttgtggattgccattgcctggtccttctaatgcaaggattacgccccgccctgccact
ajg1000	ttgcgcgggttgtcctgcgcg
ajg1001	gcatgttctgcattccctgcc
ajg1050	aaaaatcctgattcggtgtag
ajg1051	gcttatcaggcctacaatcaa
ajg1052	gcttatcaggcctacaatcaaaaggcggtcatatgaccgcctttttttattgcaacaaagcgacgcactttgcgccgaat
ajg1053	cgcgttaacaggcagttaatgcgggttagtgaacgttcactgacgagggtggactaaaacttacgccccgccctgccact

ajg1054	cgcgttaacaggcagttaatg
ajg1055	tttaaagatggggacaattca
ajg1056	ctttggttttcacgcaacgca
ajg1057	ccactcaaaatgaagcctcgt
ajg1058	ccactcaaaatgaagcctcgtgcgcttcctaagtctattgtcgcggagtttaagcagtgacgacgcactttgcgccgaat
ajg1059	cccttcagcactttgttcagttgcatttctttaattcctttagtaatcaattaattattattacgccccgccctgccact
ajg1060	cccttcagcactttgttcagt
ajg1061	cgccttcgctaccgtcattgc
ajg1062	ctttggttttcacgcaacgca
ajg1063	ccactcaaaatgaagcctcgt
ajg1064	ccactcaaaatgaagcctcgtgcgcttcctaagtctattgtcgcggagtttaagcagtgacgacgcactttgcgccgaat
ajg1065	gccaaccagtaacgacagactcaacaggtgatgtctgaagttactgctcatgcaattctcttacgccccgccctgccact
ajg1066	gccaaccagtaacgacagact
ajg1067	tgagtgacgcggtcttcgacc
ajg1068	gaataagcgggttaactataa
ajg1069	atggcgtccacatatcgcact
ajg1070	atggcgtccacatatcgcactacaataagagctaacacttaccagttcagggaaaccacacgacgcactttgcgccgaat
ajg1071	tacaggtgcagggcattattcagatcttatatgagcgggccgtcaggcccgttcacgtttttacgccccgccctgccact
ajg1072	tacaggtgcagggcattattc
ajg1073	ttgcaagcgttacgcgcttta
ajg1074	ttgcatgtgcgttggctgcac
ajg1075	tcctgaaacgtgacctattta
ajg1076	tcctgaaacgtgacctatttagagtattaaataagcagaaaagatgcttaagggatcacgcgacgcactttgcgccgaat
ajg1077	ctacggactcaggcaggtcgggaacaagaatatctacgctactcattatttatcctttatttacgccccgccctgccact
ajg1078	ctacggactcaggcaggtcgg
ajg1079	agtttcgatttctaccagcac
ajg1080	ttgcatgtgcgttggctgcac
ajg1081	tcctgaaacgtgacctattta
ajg1082	tcctgaaacgtgacctatttagagtattaaataagcagaaaagatgcttaagggatcacgcgacgcactttgcgccgaat
ajg1083	taaaggtggccaaccatgtcgaaaacggacatttatctgttcccgcaggaacagcgagttttacgccccgccctgccact
ajg1084	taaaggtggccaaccatgtcg
ajg1085	ttacaaagccaataattttat

Table S1. Primer sequences used in this study.
